# Topological liquid diode

**DOI:** 10.1126/sciadv.aao3530

**Published:** 2017-10-27

**Authors:** Jiaqian Li, Xiaofeng Zhou, Jing Li, Lufeng Che, Jun Yao, Glen McHale, Manoj K. Chaudhury, Zuankai Wang

**Affiliations:** 1 Department of Mechanical and Biomedical Engineering, City University of Hong Kong, Hong Kong, China.; 2 Science and Technology on Microsystem Laboratory, Shanghai Institute of Microsystem and Information Technology, Chinese Academy of Sciences, 865 Changning Road, Shanghai 200050, China.; 3 State Key Laboratory of Heavy Oil Processing, China University of Petroleum (East China), Qingdao 266580, China.; 4 Faculty of Engineering and Environment, Northumbria University, Newcastle upon Tyne NE1 8ST, UK.; 5 Department of Chemical Engineering, Lehigh University, Bethlehem, PA 18015, USA.; 6 Shenzhen Research Institute of City University of Hong Kong, Shenzhen 518057, China.

## Abstract

The last two decades have witnessed an explosion of interest in the field of droplet-based microfluidics for their multifarious applications. Despite rapid innovations in strategies to generate small-scale liquid transport on these devices, the speed of motion is usually slow, the transport distance is limited, and the flow direction is not well controlled because of unwanted pinning of contact lines by defects on the surface. We report a new method of microscopic liquid transport based on a unique topological structure. This method breaks the contact line pinning through efficient conversion of excess surface energy to kinetic energy at the advancing edge of the droplet while simultaneously arresting the reverse motion of the droplet via strong pinning. This results in a novel topological fluid diode that allows for a rapid, directional, and long-distance transport of virtually any kind of liquid without the need for an external energy input.

## Teaser

Janus gate: A unique topology blocks the flow of water in one direction but makes it run fast in the opposite direction.

## INTRODUCTION

Directed and spontaneous transport of a liquid on a solid surface is highly desired in various settings that range from microfluidics, printing, and oil-water separation to water harvesting technologies (*1*–*8*). Although this field of research has blossomed over the last two decades, the available technologies are far from what would be needed for controlling a desired fluidic process with a high degree of fidelity. The principal detriment to the generation of this type of fluidic motion arises from surface defects that pin the droplet edge, thus thwarting its motion. To combat the pinning forces, an external source of energy is generally needed that often mimics the rectification of the random motion of particles manifesting in various natural and man-made settings (*9*–*15*). Remarkably, many living organisms [such as the pitcher plant, cactus, and lizard (*16*–*18*)] perform the task of liquid transportation at a small scale with immaculate precision by taking advantage of surface topography alone. In recent years, extensive efforts have been directed toward mimicking these types of transport diversity that fundamentally resort to the breaking of wetting symmetry (*7*, *19*–*26*). Despite commendable progress, it remains a daunting undertaking to mimic the structural and the functional sophistication inherent in living organisms in a facile and reproducible manner.

The inherent challenge in attaining a unidirectional and continuous liquid transport stems from the fact that one edge of the droplet needs to be activated, whereas its other edge needs to remain pinned. To meet this objective, we resort to the fundamental fact that pinning barriers can be mitigated by allowing droplets to coalesce with each other near a pinning boundary (*5*, *27*, *28*). By building upon this science and taking advantage of topographical complexities, we demonstrate here that a desired unidirectional motion of the liquid droplet can be achieved by leveraging the coalescence of a pinned droplet with a thin precursor film that spreads rapidly ahead of the edge. In turn, the same topographical complexity or structure leads to a simultaneous arrest of the reverse motion of the droplet via strong pinning. The design of this novel liquid diode shifts away from the conventional paradigm in which a continuous gradient of wettability is invariably used to generate droplet motion.

## RESULTS

**Fig. 1:**
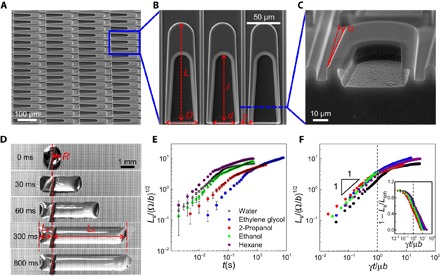
Design and characterization of liquid diode. (**A**) SEM image of the as-designed liquid diode. (**B**) Magnified SEM image of U-shaped island arrays with the reentrant cavity on one end. *L* and *D* are the length and width of the island, *l* and *d* are the length and width of the cavity, and *s* is the opening width of divergent side-channel. Here, *L* ~ 150 μm, *D* ~ 50 μm, *l* ~ 100 μm, *d* ~ 30 μm, and *s* ~ 5 μm. (**C**) Magnified cross-sectional view of the reentrant structure at the inner wall of the cavity. α is the apex angle of the diverging side-channel, and here, α ~ 2.2°. (**D**) Optical images of time-dependent directional liquid spreading on liquid diode. A water droplet (~5 μl) deposited on the surface propagates preferentially in the direction toward the opening of the cavities and gets pinned in the reverse direction. The rectification coefficient is 5.76. (**E**) The normalized plot of time-dependent liquid spreading on liquid diode. (**F**) Rescaled plot of the data summarized in (E). The droplet in the later stage exhibits a logarithmic slowing-down kinetics as evidenced in the semi-log plot of 1 − *L*_s_*/L*_sm_ versus *t/*τ (inset), where *L*_sm_ is the maximum spreading length.

Figure 1A shows the scanning electron microscope (SEM) image of the as-designed liquid diode, consisting of U-shaped island arrays spatially confined in periodically patterned fences. The width of the U-shaped islands is designed to decrease gradually from the opening end to the other end; thus, two diverging side-channels are naturally formed within fences (Fig. 1B). Moreover, as shown in Fig. 1C, the inner side of the cavity in the U-shaped island is specially designed with a reentrant structure based on inspiration of previous studies (*29*–*32*) to constrict the backflow of the liquid. The width (*d*) and length (*l*) of the cavity, the total width and length of the island (*D* and *L*), and the spacing between individual islands can be varied. All surfaces are fabricated on a silicon wafer using standard microelectromechanical system (MEMS) processes (fig. S1 and table S1). A water droplet (~5 μl) deposited on the as-fabricated surface displays an asymmetric transport behavior. As shown in Fig. 1D and movie S1, there is a dominant propagation of the liquid in the lateral direction toward the opening of the U-shaped islands, denoted as the preferential direction. In the first few milliseconds, the droplet also spreads slightly in the direction opposite to the opening of cavities (denoted as the reverse direction), which, nevertheless, becomes rapidly pinned by the convexity of the U-shaped channel. Together, these two functions display the typical signature of a directed liquid transport, which is also manifested with any other liquid including ethanol, ethylene glycol, and alkanes (Fig. 1E) that meet the Concus-Finn corner flow conditions (*33*), in which the intrinsic contact angle (θ) and the half-angle (β) of the corner in the microchannel follow the inequality condition: θ < π/2 − β. After a sufficiently long time, the elongated droplet stops spreading. By denoting the spreading length of the droplet in the major spreading direction (*L*_s_) relative to that in the pinning direction (*L*_p_), a rectification coefficient (*k*) can be defined as *L*_s_*/L*_p_ that quantifies such diode-like behavior. For the as-fabricated liquid diode shown in Fig. 1, *k* is measured to be 5.76, which is larger than that of any surfaces reported previously (*7*, *16*, *19*–*25*).

In what follows, we demonstrate that the overall spreading dynamics of various liquids on such a surface results from discrete events. Discrete flow in wetting has been previously reported. For example, Chaudhury and Chaudhury (*34*) reported an experiment about a decade ago, in which a drop of oil spreads fast through a linear array of small water droplets placed on a hydrophobic surface via repeated events of accumulation of oil under each drop, followed by its spreading to the next droplet. Although similar discrete wetting has also been observed with other systems (*7*, *20*, *22*), the crucial physics of how the spreading liquid overcomes the pinning barrier has not been discussed in the previous studies. On the basis of the observations reported below, we find that the unidirectional spreading results from the combination of events in which a precursor film spreading ahead of the bulk of the drop plays an important role. The bulk drop undergoes a hydraulic jump (*35*, *36*) because its flow is arrested at the pinning edge, which subsequently coalesces with the precursor film, thus reinitiating its spreading until the next pinning edge is reached.

In Fig.1E, we plot the time-dependent spreading length normalized by a characteristic geometric length (Ω/*b*)^0.5^ of the spreading droplet, where Ω is the volume of the droplet and *b* is its lateral width. With a modest dynamic range of data spanning over three decades, two different spreading regimes can be clearly identified. Initially, the spreading length increases almost linearly with time, suggesting that the velocity of the droplet remains more or less constant, signifying a unique characteristic of the designed liquid diode. In the linear growth regime, the higher viscosity droplet spreads more slowly than a relatively inviscid droplet. Nondimensionalization of the experimental spreading time with an intrinsic viscocapillary time scale τ = μ*b/*γ brings the scattered data for various liquids quite close to each other (Fig. 1F), which encourages us to state that the scaling *L*_s_/(Ω/*b*)^0.5^ ~ *t/*τ captures the essential physics of the unidirectional spreading behavior of the droplet during the initial stage. At the later stage, the spreading exhibits a logarithmically slowing-down kinetics as evidenced in the semi-log plot of 1 − *L*_s_*/L*_sm_ versus *t*/τ (inset of Fig. 1F), where *L*_sm_ is the maximum spreading length. Such a weak temporal evolution of its spreading length departs from the classical slowing-down behavior (*34*), which is reminiscent of various processes in nature (*37*), such as the time evolution of frictional strength, compactification of grains, magnetization relaxation in spin glasses, conductance relaxations, and diffusion under a random force.

**Fig. 2:**
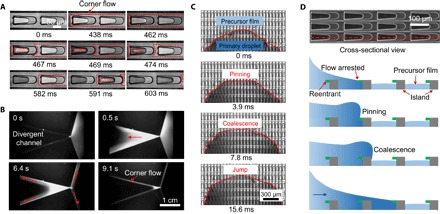
Microscopic spreading dynamics. (**A**) Schematic depiction of the corner flow induced by the side-channels and eventual filling-up of the cavities with reentrant structure. (**B**) To demonstrate the corner flow, we created two diverging channels using two nonparallel glass slides. As a water droplet containing 0.1-μm fluorescent particles is placed in the mouth of a channel (0 s), the liquid flows fast along all the available corners (indicated by the red arrow) and fills partially the entrance region of the channel. However, as flow continues along the corners, this accumulated liquid is depleted, indicating the role of the corner flow on the liquid transport. (**C**) Visualization of the time-dependent flow behavior of a water droplet on liquid diode. As the precursor film continues to flow ahead of the primary droplet, the spreading liquid accumulates to form a nearly straight wetting front as a result of contact line pinning of the advancing edge (3.9 ms). Subsequently, the primary droplet coalesces with the precursor film, and the straight edge eventually jumps like an avalanche (7.8 and 15.6 ms), resulting in a fast forward flow. (**D**) Schematic drawing showing the spreading dynamics of a primary droplet in the forward direction, which consists of pinning of the advancing edge, coalescence, and subsequent hydraulic jump with the precursor film accumulated in the reentrant cavity. The area in green corresponds to the reentrant structure.

From various observations, including that of a slowly spreading silicone fluid on such a substrate structure, it is clear that a visually perceptible thin liquid film continues to spread ahead of the primary droplet (fig. S2). Figure 2A presents time-resolved wetting dynamics of the advancing precursor liquid in two periodic units of the structure that displays the diode-like effect (movie S2). Initially, the precursor liquid preferentially wets the straight sidewall of side-channels (0 to 400 ms). Upon contact with the sidewall of U-shaped islands (428 ms), it progresses spontaneously in the side-channels, with a spreading velocity 2.8-fold larger than that of the primary droplet (fig. S3). Subsequently, the reentrant cavity and asperities between two neighboring U-shaped islands become wetted, triggering a global propagation of liquid and characteristic of a domino effect.

What appears to be a precursor film is, in fact, a consequence of a Concus-Finn type corner flow (*33*) that is generic to two wettable surfaces producing a corner. In our diode structure, such a flow manifests in the form of thin liquid threads with a smoothly varying curvature spreading along the side fences and then distributing to other available corners. The role of corner flow in dragging a liquid in a diverging channel can be easily demonstrated by placing a small droplet of water near the narrow gap of two slightly nonparallel glass slides, as shown in Fig. 2B (movie S3). Upon invading the diverging channel through the narrow gap, the water fills it up rather fast. However, as the liquid continues to spread along all the four corners of this structure, it is eventually depleted (see movie S3), illustrating that the pressure gradient in the liquid along the corners is stronger than the Laplace pressure gradient that is produced across the concave meniscus of the liquid joining the two walls of a diverging channel.

To elucidate how the corner flow in the precursor regulates the unusual unidirectional spreading behavior, we further analyze the local contact line dynamics of the primary droplet. We find that the advancing edge of the primary droplet does not move continuously. Instead, it stops for a brief period during which the precursor film continues to flow. When the bulk flow of the liquid drop is suddenly arrested, Bernoulli’s equation suggests that the conserved energy of the liquid has to be converted to another form of energy. This happens in our case with the liquid bulging up near the arrested edge (the semicircular edge, Fig. 2C and movie S4), thus creating more surface area and, consequently, more surface free energy. This bulging up leads to a hydraulic jump (*35*, *36*) that results in an increase of the local contact angle. The pinned edge moves forward when the local contact angle increases above a certain critical value dictated by the intrinsic contact angle and the slope of pinning edge. As different segments of the pinned edge are successively depinned, a nearly straight frontal edge is formed, following which the entire liquid front jumps forward like an avalanche (Fig. 2D). Because the advancing edge relaxes from a semicircle of diameter ~ *b*, the characteristic time scale of this process can be written as τ *=* μ*b/*γ. For a droplet with a constant volume (Ω) and a lateral spreading width *b*, the intrinsic length of spreading is (Ω/*b*)^0.5^. Thus, the scaling for the spreading distance in the fast spreading regime becomes *L*_s_/(Ω/*b*)^0.5^ ~ *t/*τ, which is consistent with the results summarized in Fig. 1F. In closing, we believe that the hydraulic jump rendered by the corner flow provides a unique mechanism for the droplet to overcome the contact line pinning in the preferential flow direction.

**Fig. 3:**
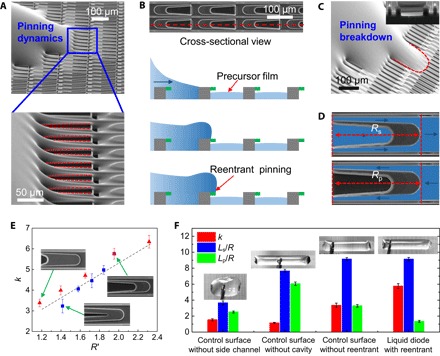
Microscopic pinning dynamics and quantification of rectification coefficient. (**A**) SEM images showing the pinning of advancing liquid at the reentrant structure (top). The red lines in the magnified SEM image (below) denote the pinning of the liquid at the reentrant edge. (**B**) Schematic drawing of the effect of reentrant structure (in green) on the liquid penetration. In conjunction with the concave meniscus in the diverging channel, the advancing edge in the primary droplet fails to coalesce with the precursor liquid. (**C**) SEM image of the breakdown of the liquid pinning on control surface without the reentrant structure. It is clear that the advancing liquid penetrates the cavity, and the pinning effect is collapsed as depicted by the red dashed line. The inset shows the side view of the cavity with a straight sidewall. (**D**) Schematic drawing of the flow pathways in spreading and pinning directions. (**E**) Plot of the rectification coefficient (*k*) versus *R*′, the ratio of hydraulic resistances between two different directions. Here, the *R*′ can be tailored by varying sizes in the cavity length or width. The red triangles indicate a series of surfaces with increasing cavity length (^*l*^/_*L*_ = ^1^/_6_, ^1^/_3_, ^1^/_2_, ^2^/_3_, and ^5^/_6_), but with constant width (^*d*^/_*D*_ = ^3^/_5_), as shown in table S2 and fig. S8A. The blue squares indicate a series of surfaces with increasing cavity width (^*d*^/_*D*_ = ^1^/_5_, ^3^/_10_, ^2^/_5_, ^1^/_2_, and ^3^/_5_), but with constant length (^*l*^/_*L*_ = ^2^/_3_), as shown in table S3 and fig. S8B. (**F**) Comparison of the transport performances between different surfaces. The blue and green symbols indicate the spreading lengths normalized by the droplet radius at the spreading direction and pinning direction, respectively. The red symbol denotes the rectification coefficient (*k*) on different designs. The inset images present the optical image of spreading drops on the respective surfaces.

Despite the corner flow and hydraulic jump, the backflow of the liquid droplet is strongly thwarted around the reentrant edge (*29*, *30*), as evidenced by the SEM image of the contact line in Fig. 3A (figs. S4 and S5). As shown in Fig. 3B, the presence of unique reentrant structure gives rise to an upward capillary force to resist the liquid penetration into the cavity. Such a pinning is also assisted kinetically by the continual removal of liquid through the corner flow in the diverging channel, as the curvature of the liquid meniscus becomes progressively concave starting from the mouth of the diverging channel toward its tail. Collectively, these two effects nullify the possibility of the droplet relaxing from the pinned edge. However, for the surface without the presence of reentrant structure (inset of Fig. 3C), the advancing liquid in the pinning direction easily penetrates the cavity, and the pinning effect is broken down, as depicted by the continuous advancing edge shown in Fig. 3C (fig. S6). In summary, the spreading droplet can only flow in one, but not its reverse, direction.

The decoupling of the side-channels responsible for the corner flow from the reentrant cavities responsible for the pinning of backflow creates distinct hydrodynamic flow resistances (*38*) in the spreading and pinning directions (Fig. 3D and fig. S7). In the spreading direction, the fluid propagates along both side-channels and cavities. By contrast, in the reverse direction, the pathway is confined to side-channels alone owing to the reasons discussed above. When the droplet spreading comes to a complete halt, an interesting relation emerges, which connects the asymmetric spreading lengths to the hydraulic flow resistances in two directions. Such a unique relation arises from the fact that the rate of the decay of the droplet volume is exactly the same as that carried outward by the precursor films in two opposite directions. The flow in the precursor film is mainly driven by the negative pressure (−P*) in the precursor film, − *P* * ~ *R*_s_*Q*_s_ ~ *R*_p_*Q*_p_, where *R*_i_ and *Q*_i_ = *bL*_i_(*dh*/*dt*) are the hydrodynamic flow resistance and the volumetric flow rate either in the spreading (*i = s*) or the pinned (*i = p*) direction. Combining these two conditions, we have *R*_s_*L*_s_ ~ *R*_p_*L*_p_ and, hence, *k* ~ *R*_*p*_/*R*_*s*_. On the other hand, the hydraulic flow resistance in the reverse direction relative to that in the spreading direction can be written as *R*′ = *R*_p_/*R*_s_ = 1 + *R*_side_/2*R*_cavity_, where *R*_side_ and *R*_cavity_ are the hydraulic resistances in the side-channel and cavity, respectively. In conformity with the above scenario, we find that the rectification coefficient is linearly proportional to *R*′ (Fig. 3E and figs. S8 and S9). Thus, tailoring the feature size of cavity alone (tables S2 and S3) allows the regulation of the rectification coefficient in the liquid diode without compromising the fast and spontaneous liquid transport in the side-channels.

To ascertain how the preferential motion of droplet is mediated by structural topography, we also designed and fabricated control surfaces without the presence of a side-channel, cavity, or reentrant structure (Fig. 3F and table S1). The results show that the manifestation of a superior diode-like behavior demands the delicate control over the diverging channel, cavity, and reentrant structure. Briefly, with the obstruction of the side-channel or the cavity, both the spreading length of droplet and rectification coefficient are markedly reduced (Fig. 3F). For the control surface without the presence of side-channels (fig. S10A and table S1), the droplet displays an almost symmetric spreading with a short spreading distance in all directions (fig. S11A). This result suggests that corner flow serves as an energetically favorable pathway to guide the lateral liquid flow. For the control surface in which the cavity is obstructed (fig. S10B and table S1), the droplet spreading is spatially confined in the lateral direction (fig. S11B). However, in this case, the rectification coefficient is the smallest (Fig. 3F), suggesting that the cavity structure is essential to the rectification of directional flow. Moreover, without the presence of reentrant structure in the cavity (fig. S10C), the rectification coefficient is reduced by about 40% (Fig. 3F and fig. S11C). Together, these results convincingly reveal that the exquisite integration of diverging side-channels (corner flow) and reentrant in one structure (hydraulic jump and reentrant pinning) is indispensable for the directional and fast liquid transport.

## DISCUSSION

**Fig. 4:**
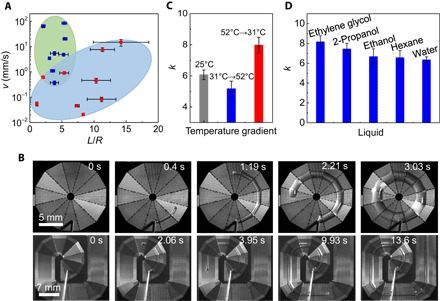
Generality of the liquid diode. (**A**) Comparison of transport performances among different surfaces. The green area and the blue area indicate the surfaces with wettability gradients and asymmetric geometries, respectively (see fig. S12). The red triangle denotes the unidirectional liquid transport on the natural peristome of a pitcher plant. The red circle represents the as-designed liquid diode. (**B**) Continuous and directional water transportation on surfaces with circular and spiral pathways. (**C**) Variation of the rectification coefficient under different temperature gradients, showing the stability of the directional liquid transport. (**D**) Generality of directional liquid transport for all kinds of liquids. The liquid diode exhibits a high rectification coefficient for low–surface tension liquid, such as hexane, and high-viscosity liquid, such as ethylene glycol.

To illustrate the advantages of our liquid diode, in Fig. 4A, we benchmark the unidirectional spreading velocity and the range of transport with reported surfaces (fig. S12). Surfaces with an imposed wetting gradient (*19*, *25*, *39*–*44*) are associated with a large motion velocity (green area in Fig. 4A) and a short spreading distance owing to the inherent tradeoff in satisfying these two exclusive parameters. On the other hand, asymmetrically structured surfaces (*7*, *16*, *20*–*22*, *45*, *46*) enable a long range of liquid self-transport, but with a small velocity (blue area in Fig. 4A). We also demonstrate that the intriguing liquid transport on the as-designed liquid diode is general. First, as shown in Fig. 4B, the liquid can be transported along various pathways (such as rings and circles) and can climb uphill without the use of gravity or other gradient (movies S5 and S6). Second, as shown in Fig. 4C, the unidirectional liquid transport is further enhanced when there is a gradual temperature gradient (fig. S13) along the spreading direction. When the temperature gradient is applied against the spreading direction, there is only a slight decrease in the rectification coefficient, suggesting the stability of the directional transport even when subject to an externally applied temperature field. Such a capability holds many potential applications, such as in heat pipes (*47*). Finally, the directional droplet transport applies to any liquid including low–surface tension liquid, such as hexane (~18.4 mN/m), and high-viscosity liquid, such as ethylene glycol (~16.06 mPa·s) (Fig. 4D and table S4). However, the high-viscosity liquid (ethylene glycol) displays a counterintuitive behavior in which its rectification coefficient is higher than that of water.

The criterion of rectification as discussed above is based on uniform reduction in the thickness of the bulk of the liquid when spreading comes to a complete halt. With more viscous liquid, the rate of thinning of the liquid film in the bulk of the droplet should be spatially uneven, because another resistance corresponding to the horizontal internal flow of the liquid drop needs to be considered. Although a rigorous solution of this problem requires a full consideration of the so-called “thin-film” equation (*36*), we expect the bulk flow of the liquid to occur (internally) from the pinned direction to the spreading direction of the drop, such that $\frac{dh_{p}}{dt}>\frac{dh_{s}}{dt}$. Because the rectification coefficient would be of the form $k=(R_{p}\frac{dh_{p}}{dt})/(R_{s}\frac{dh_{s}}{dt})$, we expect *k* to increase for a viscous liquid in comparison to that of a more inviscid liquid (Fig. 4D).

Together, the ease of fabrication, generality, and intriguing transport performances of our liquid diode will open up new avenues to tailor advanced microstructures for self-propelled liquid manipulation in a variety of applications, including water harvesting, heat management, ink-jet printing, and emulsion separation.

## MATERIALS AND METHODS

### Sample fabrication

We used standard MEMS processes to fabricate the liquid diode based on silicon wafer [(100) type, 420 ± 5 μm thick]. To form side-channels, we first used the photolithography process to deposit a layer of photoresist as a protective mask, followed by anisotropic etching (~5 μm deep) using deep reactive ion etching (DRIE). Subsequently, plasma strip and wet cleaning were performed to remove the photoresist. To fabricate the cavities with reentrant structure, we used thermal oxidization to deposit a layer of SiO_2_ with a thickness of 1 μm on the silicon wafer, followed by photolithographic patterning and selective removal of the SiO_2_ layer using reactive ion etching. The silicon exposed at the cavity structure was isotropically etched (~5 μm deep) by DRIE or XeF_2_. After final wet cleaning treatment, the liquid diode with reentrant structure was formed.

To investigate the effect of surface topography on the liquid diode behavior, we also fabricated a series of surfaces with varying sizes of cavity structure and side-channels, as well as control surfaces without the design of cavity, reentrant, or side-channel. The control surface without the cavity structure was fabricated with the first three steps of the process described above. The procedure to fabricate the control surface without the reentrant structure is basically similar to that developed for the fabrication of the liquid diode with a reentrant feature, except that the Si was anisotropically etched to form the cavity with a straight sidewall. The control surface without side-channels was fabricated using the same procedure as developed for the liquid diode surface, except that the masks for photolithography in the first step were different. Compared to the liquid diode surface, the side-channel was blocked on the control surface without the design of side-channel. We also designed liquid diodes with spiral or circular pathways to demonstrate the generality of the directional transport. To fabricate the curved pathways, we divided the surfaces into a variety of parts, and each part was fabricated by the procedures developed for the liquid diode surface with the reentrant structure. Note that owing to the existence of defects between neighboring parts, the flow widened slightly during the directional liquid transport on the surface with spiral or circular pathways.

### Surface treatment and contact angle measurement

We treated the as-fabricated surfaces and the flat silicon substrate with Plasma Cleaner PDC-32G (Harrick Plasma) at high radio frequency levels for ~90 s to obtain hydrophilic surfaces. To quantify how the intrinsic wettability affects the droplet spreading behavior, we measured the intrinsic contact angle on the flat surface using a contact angle goniometer (ramé-hart M200 Standard Contact Angle Goniometer). At room temperature with 50% relative humidity, a deionized water droplet of ~3 μl was deposited on the tested substrates at a volume rate of 1 μl/s. We also performed contact angle measurements every 10 min for a total of 1 hour to evaluate the variation of contact angle. The apparent contact angle on the flat wafer surface within an hour remained 14.7° ± 3.3°. These values are the average of five measurements.

### Characterization of macroscopic transport behavior of liquid diode

We performed the unidirectional spreading experiment on the as-fabricated liquid diode and control surfaces at room temperature. The liquid droplet was generated with a stainless steel needle having an outer diameter of 0.5 mm, which was connected to a syringe pump. The spreading dynamics was recorded from the plan view by a high-speed camera (FASTCAM SA4, Photron). Next, to demonstrate the generality of the directional transport, we performed liquid transport experiments on the liquid diode surfaces with circular and spiral pathways in which the water was continuously infused on these surfaces. Finally, four other liquids were also used to demonstrate the generality of the directional liquid transport.

### Characterization of microscopic transport behavior of liquid diode

To improve the visual clarity and the spatial resolution of water spreading on the liquid diode, we stained the deionized water (about 1 μl) with a blue dye. The spreading dynamics on the liquid diode was observed with an Olympus BX60 microscope connected to a high-speed camera. To examine the pinning dynamics on the liquid diode and the control surface without reentrant structure, we used a long working distance microscope equipped with a high-speed camera.

Technically, it is extremely challenging to capture the pinning profiles induced by the reentrant structure because the deionized water easily evaporates in the vacuum chamber of a SEM. To overcome this issue, we used uncured polydimethylsiloxane (PDMS) to replace water. We first deposited PDMS liquid on the liquid diode surface and then immediately heated it at 100°C for ~40 min. After a layer of silver was plated on the tested surfaces by a sputtering machine, we characterized the pinning morphology under a SEM. Likewise, we used this method to characterize the collapse of pinning effect on the control surface without the presence of reentrant structure.

### Effect of temperature gradient on the directional transport

To examine the stability of directional, spontaneous transport manifested by the liquid diode, we imposed an additional temperature gradient. Briefly, one end of the liquid diode surface was fixed onto a heater, and the other end was exposed to air. The distribution of temperature was measured using an infrared camera with a 13-mm lens. Simultaneously, the spreading dynamics of the liquid was recorded with a high-speed camera.

## Supplementary Material

http://advances.sciencemag.org/cgi/content/full/3/10/eaao3530/DC1

## SUPPLEMENTARY MATERIALS

Supplementary material for this article is available at http://advances.sciencemag.org/cgi/content/full/3/10/eaao3530/DC1 section S1. Characterization of microscopic spreading behavior of the precursor on liquid diodes section S2. Comparison of liquid pinning behavior on liquid diode and control surfaces section S3. Flow hydraulic resistance analysis section S4. Characterization of macroscopic spreading dynamics on control surfaces section S5. Comparison of liquid self-transportation on various surfaces section S6. Temperature gradient effect on the liquid diode fig. S1. Sample fabrication. fig. S2. Characterization of precursor film and water droplet spreading velocity. fig. S3. Selected snapshots showing the microscopic wetting dynamics on the liquid diode. fig. S4. Representative SEM images showing the liquid pinning rendered by the reentrant structure. fig. S5. Selected snapshots showing the pinning dynamics of water on the liquid diode. fig. S6. Selected snapshots showing the breakdown of pinning on the control surface without the presence of a reentrant feature. fig. S7. Schematic diagrams showing the flow pathways on the control surface without the presence of a cavity. fig. S8. Effects of structural topography on the flow resistance parameter *R*′. fig. S9. Effects of structural topography on the rectification coefficient *k*. fig. S10. SEM characterization of control surfaces. fig. S11. Spreading dynamics on the control surfaces. fig. S12. Comparison of transport performances among different surfaces. fig. S13. Effect of temperature gradient on the directional transport. table S1. Structural parameters of the liquid diode and control surfaces. table S2. Structural parameters of liquid diodes with varying sizes in cavity length. table S3. Structural parameters of liquid diodes with varying sizes in cavity width. table S4. Physical and chemical properties of tested liquids. movie S1. Unidirectional spreading of a single water droplet. movie S2. Microscopic wetting dynamics on the liquid diode. movie S3. Corner flow in the divergent channel. movie S4. Hydraulic jump mechanism on the liquid diode. movie S5. Directed water transportation on circular surface. movie S6. Directed water transportation on spiral surface.
