# Supplementary material for: Topological liquid diode
Source: Sci Adv. 2017 Oct 27;3(10):eaao3530. doi: 10.1126/sciadv.aao3530 (PMC5659653; doi:10.1126/sciadv.aao3530)
Supplement: http://advances.sciencemag.org/cgi/content/full/3/10/eaao3530/DC1 [file supp_3_10_eaao3530__index.html]

Science Advances | Science Advances

## Supplementary Materials

**This PDF file includes:**

- section S1. Characterization of microscopic spreading behavior of the precursor on liquid diodes
- section S2. Comparison of liquid pinning behavior on liquid diode and control surfaces
- section S3. Flow hydraulic resistance analysis
- section S4. Characterization of macroscopic spreading dynamics on control surfaces
- section S5. Comparison of liquid self-transportation on various surfaces
- section S6. Temperature gradient effect on the liquid diode
- fig. S1. Sample fabrication.
- fig. S2. Characterization of precursor film and water droplet spreading velocity.
- fig. S3. Selected snapshots showing the microscopic wetting dynamics on the liquid diode.
- fig. S4. Representative SEM images showing the liquid pinning rendered by the reentrant structure.
- fig. S5. Selected snapshots showing the pinning dynamics of water on the liquid diode.
- fig. S6. Selected snapshots showing the breakdown of pinning on the control surface without the presence of a reentrant feature.
- fig. S7. Schematic diagrams showing the flow pathways on the control surface without the presence of a cavity.
- fig. S8. Effects of structural topography on the flow resistance parameter *R*′.
- fig. S9. Effects of structural topography on the rectification coefficient *k*.
- fig. S10. SEM characterization of control surfaces.
- fig. S11. Spreading dynamics on the control surfaces.
- fig. S12. Comparison of transport performances among different surfaces.
- fig. S13. Effect of temperature gradient on the directional transport.
- table S1. Structural parameters of the liquid diode and control surfaces.
- table S2. Structural parameters of liquid diodes with varying sizes in cavity length.
- table S3. Structural parameters of liquid diodes with varying sizes in cavity width.
- table S4. Physical and chemical properties of tested liquids.
- Legends for movies S1 to S6

Download PDF

**Other Supplementary Material for this manuscript includes the following:**

- movie S1 (.avi fomat). Unidirectional spreading of a single water droplet.
- movie S2 (.avi fomat). Microscopic wetting dynamics on the liquid diode.
- movie S3 (.avi fomat). Corner flow in the divergent channel.
- movie S4 (.avi fomat). Hydraulic jump mechanism on the liquid diode.
- movie S5 (.avi fomat). Directed water transportation on circular surface.
- movie S6 (.avi fomat). Directed water transportation on spiral surface.

**Files in this Data Supplement:**

- Adobe PDF - aao3530\_SM.pdf
