# Supplementary material for: Topological liquid diode
Source: Sci Adv. 2017 Oct 27;3(10):eaao3530. doi: 10.1126/sciadv.aao3530 (PMC5659653; doi:10.1126/sciadv.aao3530)
Supplement: http://advances.sciencemag.org/cgi/content/full/3/10/eaao3530/DC1 [file aao3530_SM.pdf]

Science Advances

AAAS

advances.sciencemag.org/cgi/content/full/3/10/eaao3530/DC1

Supplementary Materials for

Topological liquid diode

Jiaqian Li, Xiaofeng Zhou, Jing Li, Lufeng Che, Jun Yao, Glen McHale, Manoj K. Chaudhury, Zuankai Wang

Published 27 October 2017, *Sci. Adv.* **3**, eaao3530 (2017)
DOI: 10.1126/sciadv.aao3530

The PDF file includes:

- section S1. Characterization of microscopic spreading behavior of the precursor on liquid diodes
- section S2. Comparison of liquid pinning behavior on liquid diode and control surfaces
- section S3. Flow hydraulic resistance analysis
- section S4. Characterization of macroscopic spreading dynamics on control surfaces
- section S5. Comparison of liquid self-transportation on various surfaces
- section S6. Temperature gradient effect on the liquid diode
- fig. S1. Sample fabrication.
- fig. S2. Characterization of precursor film and water droplet spreading velocity.
- fig. S3. Selected snapshots showing the microscopic wetting dynamics on the liquid diode.
- fig. S4. Representative SEM images showing the liquid pinning rendered by the reentrant structure.
- fig. S5. Selected snapshots showing the pinning dynamics of water on the liquid diode.
- fig. S6. Selected snapshots showing the breakdown of pinning on the control surface without the presence of a reentrant feature.
- fig. S7. Schematic diagrams showing the flow pathways on the control surface without the presence of a cavity.
- fig. S8. Effects of structural topography on the flow resistance parameter R'.
- fig. S9. Effects of structural topography on the rectification coefficient k.
- fig. S10. SEM characterization of control surfaces.
- fig. S11. Spreading dynamics on the control surfaces.

- fig. S12. Comparison of transport performances among different surfaces.
- fig. S13. Effect of temperature gradient on the directional transport.
- table S1. Structural parameters of the liquid diode and control surfaces.
- table S2. Structural parameters of liquid diodes with varying sizes in cavity length.
- table S3. Structural parameters of liquid diodes with varying sizes in cavity width.
- table S4. Physical and chemical properties of tested liquids.
- Legends for movies S1 to S6

Other Supplementary Material for this manuscript includes the following:
(available at advances.sciencemag.org/cgi/content/full/3/10/eaao3530/DC1)

- movie S1 (.avi fomat). Unidirectional spreading of a single water droplet.
- movie S2 (.avi fomat). Microscopic wetting dynamics on the liquid diode.
- movie S3 (.avi fomat). Corner flow in the divergent channel.
- movie S4 (.avi fomat). Hydraulic jump mechanism on the liquid diode.
- movie S5 (.avi fomat). Directed water transportation on circular surface.
- movie S6 (.avi fomat). Directed water transportation on spiral surface.

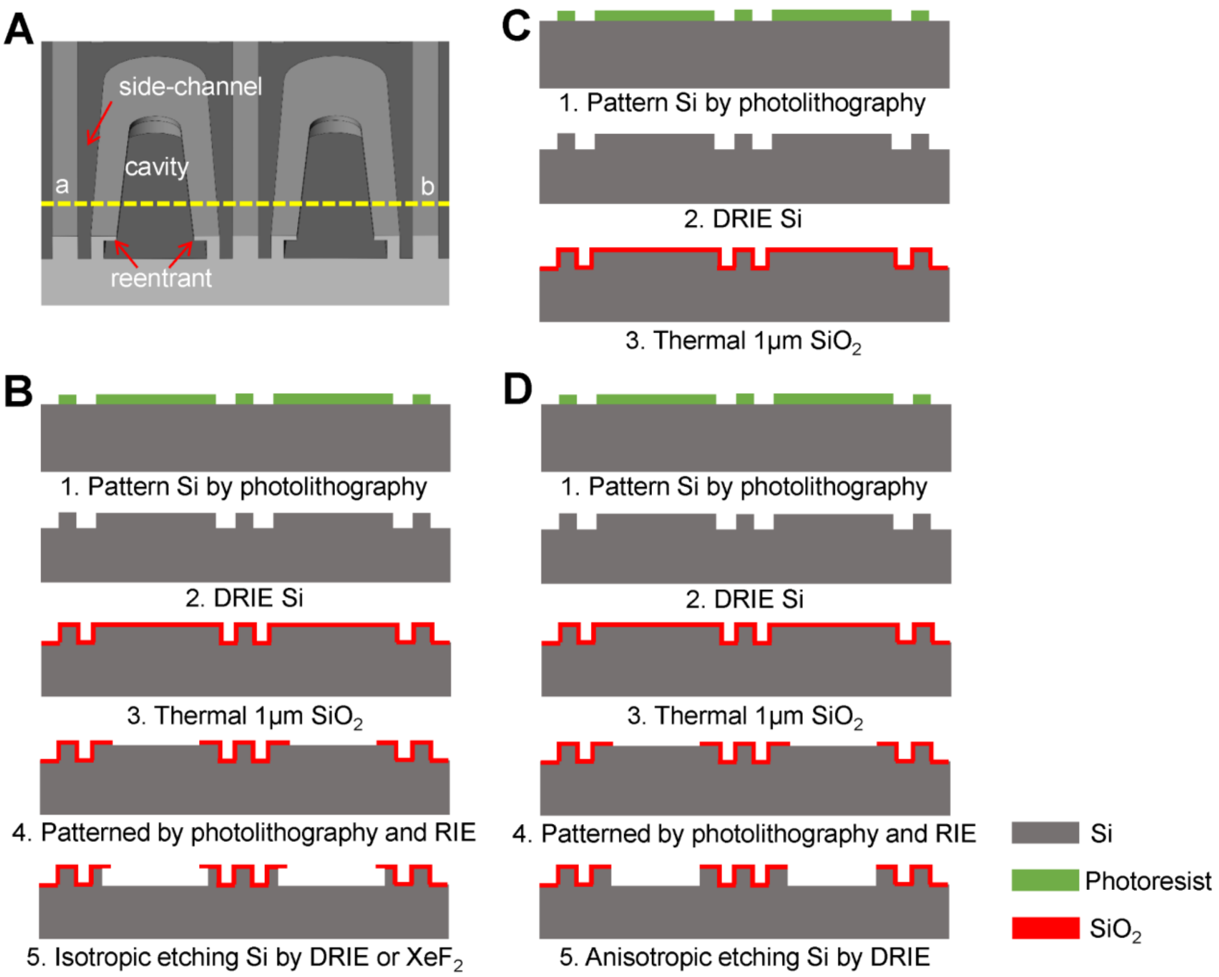

fig. S1. Sample fabrication. (**A**) Schematic diagram of liquid diode which consists of three essential structural features: side-channel, cavity and reentrant at the edge of the cavity. The yellow dashed line indicates the cross-section view to display the fabrication procedures. (**B**) Process flow to fabricate the liquid diode. (**C**) Process flow to fabricate the control surface without the presence of cavity. (**D**) Process flowchart to fabricate the control surface without the presence of reentrant structure. The gray area, green area, and red area indicate Si, photoresist and SiO_2, respectively.

table S1. Structural parameters of the liquid diode and control surfaces.

Features / Samples	s (μm)	d (μm)	l (μm)	Reentrant length (μm)
Liquid diode with reentrant	5	30	100	5
Control surface without side-channel	0	30	100	5
Control surface without cavity	5	0	0	0
Control surface without reentrant	5	30	100	0

Note: All the surfaces have same structural parameters L ~ 150μm, D ~ 50μm and α ~ 2.2°.

table S2. Structural parameters of liquid diodes with varying sizes in cavity length.

Features / Samples	*s* (μm)	*d* (μm)	*l* (μm)	Reentrant length (μm)
1	5	30	25	5
2	5	30	50	5
3	5	30	75	5
4	5	30	100	5
5	5	30	125	5

Note: All the surfaces have same structural parameters L ~ 150μm, D ~ 50μm and α ~ 2.2º.

table S3. Structural parameters of liquid diodes with varying sizes in cavity width.

Features / Samples	*s* (μm)	*d* (μm)	*l* (μm)	Reentrant length (μm)
1	5	10	100	5
2	5	15	100	5
3	5	20	100	5
4	5	25	100	5
5	5	30	100	5

Note: All the surfaces have same structural parameters L ~ 150μm, D ~ 50μm and α ~ 2.2º.

table S4. Physical and chemical properties of tested liquids.

Properties / Liquids	Chemical formula	Surface tension (mN/m)	Viscosity (mPa·s)	Density (kg/m^3)
Hexane	C_6H_{14}	18.4	0.3	660.6
2-Propanol	C_3H_8O	21.2	2.04	780.9
Ethanol	C_2H_6O	22.3	1.074	789
Ethylene glycol	$C_2H_6O_2$	48.2	16.06	1113.5
Deionized Water	H_2O	72.8	0.89	997

Note: Properties are collected at 20ºC and from CRC reference source.

section S1. Characterization of microscopic spreading behavior of the precursor on liquid diodes

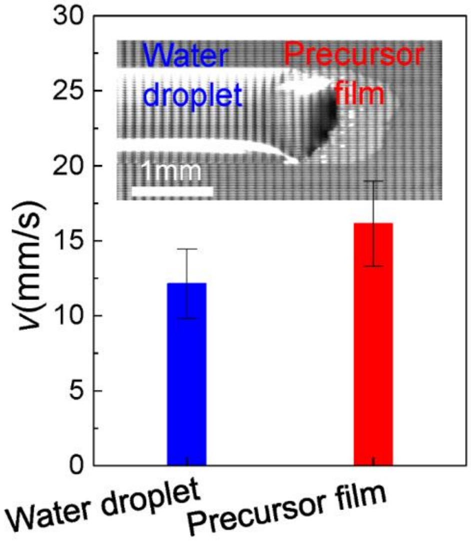

fig. S2. Characterization of precursor film and water droplet spreading velocity. During the unidirectional spreading process, there is a layer of precursor film preceding the entire water droplet. The inset image presents the selected snapshot of advancing precursor film followed by the entire water droplet.

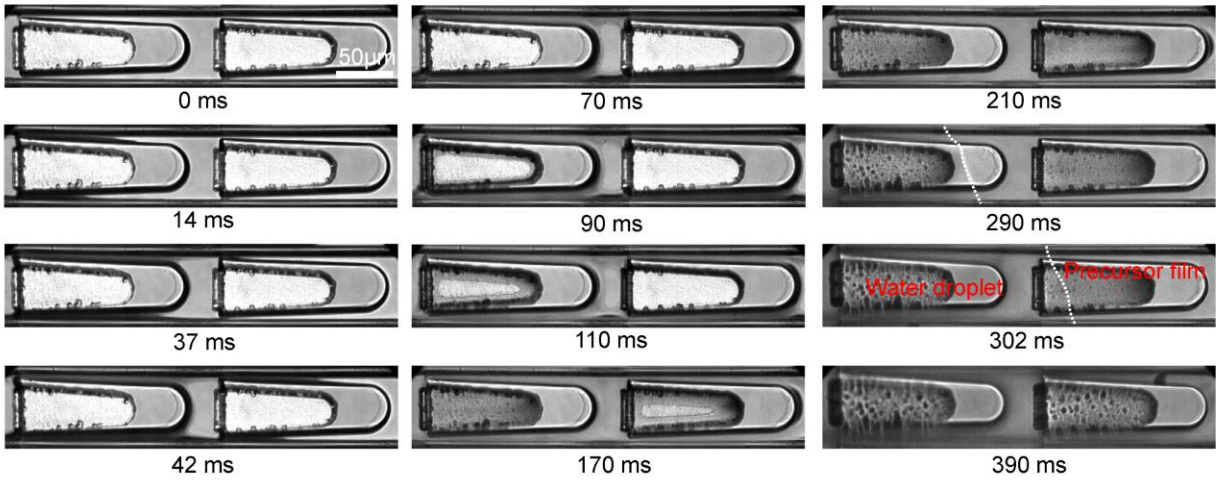

fig. S3. Selected snapshots showing the microscopic wetting dynamics on the liquid diode. When the opening width of the side channel decreases to s ~ 5 μm, the liquid filaments immediately fill the divergent side-channel (from 0 ms to 70 ms) that subsequently fill the cavities. After 210 ms, the advancing precursor precedes the entire water droplet. Here, the white dashed lines denote the moving front of the entire droplet.

section S2. Comparison of liquid pinning behavior on liquid diode and control surfaces

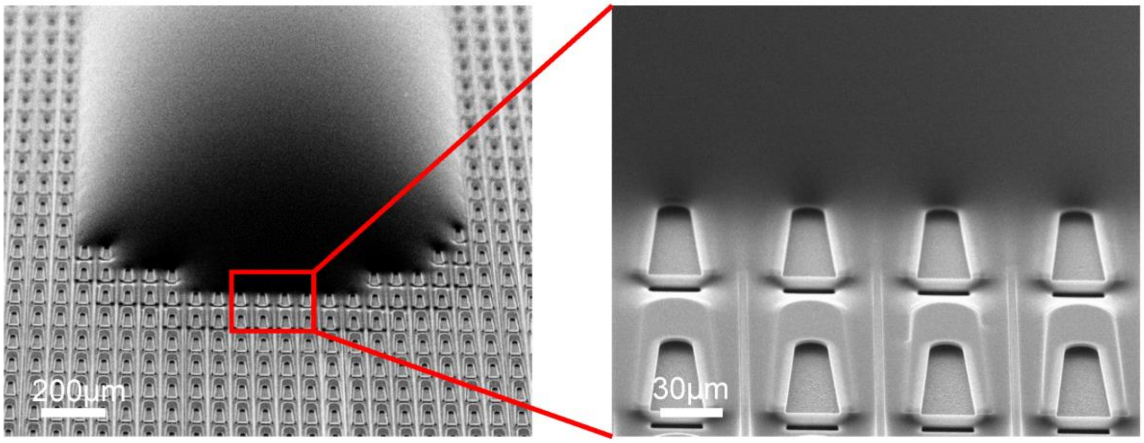

fig. S4. Representative SEM images showing the liquid pinning rendered by the reentrant structure. From the top-view image captured, it is obvious that the cured PDMS is stably immobilized around the edge of cavity owing to the pinning effect enabled by the reentrant structure.

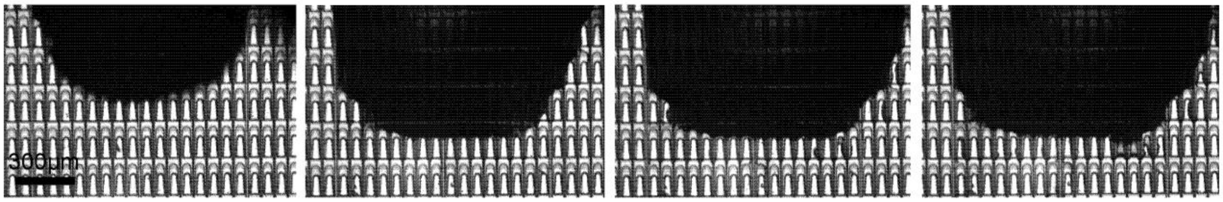

fig. S5. Selected snapshots showing the pinning dynamics of water on the liquid diode. The presence of reentrant structure distorts the curvature of the spreading liquid, preventing it from penetrating into the downsides of cavity. Although the droplet also spreads slightly towards the direction against the opening of the cavities, the spreading in the reverse direction is arrested and eventually displays a unidirectional flow along the preferential direction.

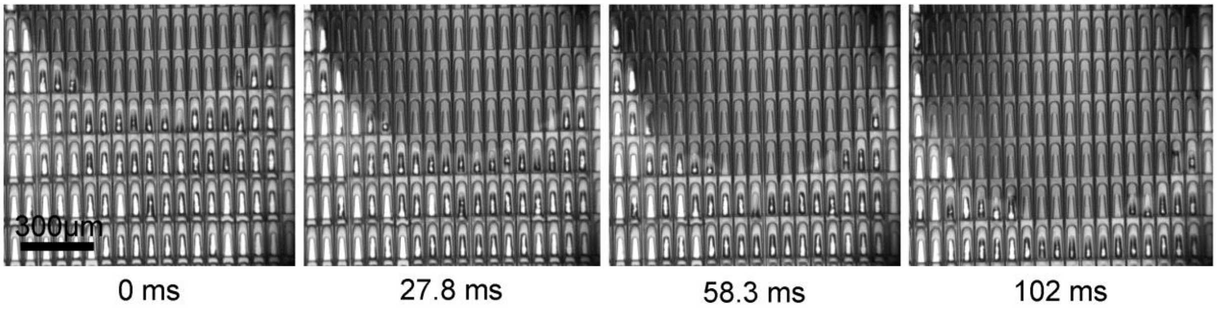

fig. S6. Selected snapshots showing the breakdown of pinning on the control surface without the presence of a reentrant feature. Because of the absence of reentrant structure around the edge of cavities, the fluid easily wets the vertical wall of the hydrophilic cavity, and exhibits a smaller asymmetric spreading in the lateral direction.

section S3. Flow hydraulic resistance analysis

To build the link between surface topography and liquid diode transport behavior, we developed a theoretical model based on the flow hydraulic resistance. We first assumed that the flow in the rectangular microchannel is fully developed. Under this condition, the hydraulic resistance in the microchannel with rectangular cross-section (width *w*, height *h* and length *l*) can be expressed as

$$R_{\text{hyd}} = \frac{12\mu l}{wh^3}\left(1 - \frac{192h\Sigma}{\pi^5 w}\right)^{-1} \tag{S1}$$

where $\Sigma = \sum_{i=1,3,5,\ldots}^{\infty} \tanh(i\pi w/2h)/i^5$.

Consider two straight channels that are connected in parallel. The hydraulic resistance across the parallel channels R_{parallel} can be characterized as

$$R_{\text{parallel}} = \left(1/R_1 + 1/R_2\right)^{-1} \tag{S2}$$

where R_1 and R_2 are the hydraulic resistances in two parallel channels, respectively. However, in the case of the serial coupling, the coupling hydraulic resistance R_{series} is calculated as

$$R_{\text{series}} = R_1 + R_2 \tag{S3}$$

where R_1 and R_2 are the hydraulic resistances in two serial channels, respectively. Along the preferential direction, the liquid spreads across two side-channels and asymmetric cavity. The hydraulic resistance along the preferential direction is expressed as

$$R_{\text{s}} = (2/R_{\text{side}} + 1/R_{\text{cavity}})^{-1} \tag{S4}$$

Along the pinning direction, however, the pathway is confined to side-channels alone owing to the presence of reentrant structure, which gives rise to a large breakthrough pressure for the liquid to penetrate. So the hydraulic resistance along the pinning direction is calculated as

$$R_{\text{p}} = R_{\text{side}}/2 \tag{S5}$$

Here R_{side} and R_{cavity} are the flow resistances in the side-channel and cavity, respectively. In addition, on the control surface without the use of cavity structure, the hydraulic resistances along the spreading direction and the pinning direction are definitely identical (fig. S7).

To build a link between surface topography and the liquid diode behavior, we defined a dimensionless ratio parameter R' of flow resistance at the pinning direction to flow resistance at the spreading direction just across single U-shaped island unit.

$$R' = R_{\text{p}} \big/ R_{\text{s}} = 1 + R_{\text{side}} \big/ 2R_{\text{cavity}} \tag{S6}$$

In order to numerically calculate the flow resistance and the parameter R', we developed a MATLAB code based on the above derivation with the following steps:

(1) The diverging side-channel and converging cavity have rectangular cross-sections, but their cross-sections vary with the spatial location x. Set the unit channel length dl and assume that the rectangular cross-sections within unit length dl are constant.

(2) Give the data of channel widths for side-channel $w_{\text{side}}(x)$ and cavity $w_{\text{cavity}}(x)$ within single U-shaped island unit based on the specified unit length dl.

(3) Compute the hydraulic resistance function for side-channel and cavity based on corresponding channel width $w(x)$, height h and unit length dl.

(4) According to derivation in this section, couple all the channels in parallel or in series to calculate the hydraulic resistance R_{s} and R_{p}, and finally compute the value of the parameter R'.

Based on the above ideas, the value of the dimensionless parameter R' can be calculated by the following formula

$$R' = \frac{\sum\limits_{x\in(0,l)} \dfrac{2\Lambda}{\left(2w_{\text{side}}(x) + w_{\text{cavity}}(x)\right)} + \sum\limits_{x\in(l,L)} \dfrac{\Lambda}{w_{\text{side}}(x)}}{\sum\limits_{x\in(0,L)} \dfrac{\Lambda}{w_{\text{side}}(x)}} \tag{S7}$$

where $\Lambda = \dfrac{6\mu dl}{h^3\left(1 - 192h\Sigma \big/ \pi^5 w\right)}$.

Finally, we investigated the correlation between surface topography and *R*'. The R' can be tailored by varying sizes in the cavity length or width (fig. S8), which regulate the rectification coefficient on the liquid diodes (fig. S9). Interestingly, experimental results based on the liquid diodes with varying topographies reveal that *k* is linearly proportional to *R*' (Fig. 3E). Thus, the rectification coefficient can be regulated by changing the relative size of cavity without affecting the liquid transport.

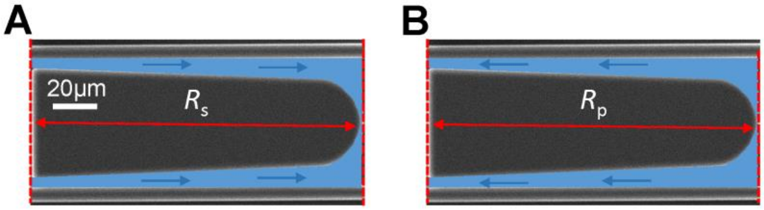

fig. S7. Schematic diagrams showing the flow pathways on the control surface without the presence of a cavity. (**A**) The hydraulic resistance R_s at the spreading direction. (**B**) The hydraulic resistance R_p at the pinning direction. Blue area denotes the liquid in the channels and the dark blue arrows denote the direction of the flow.

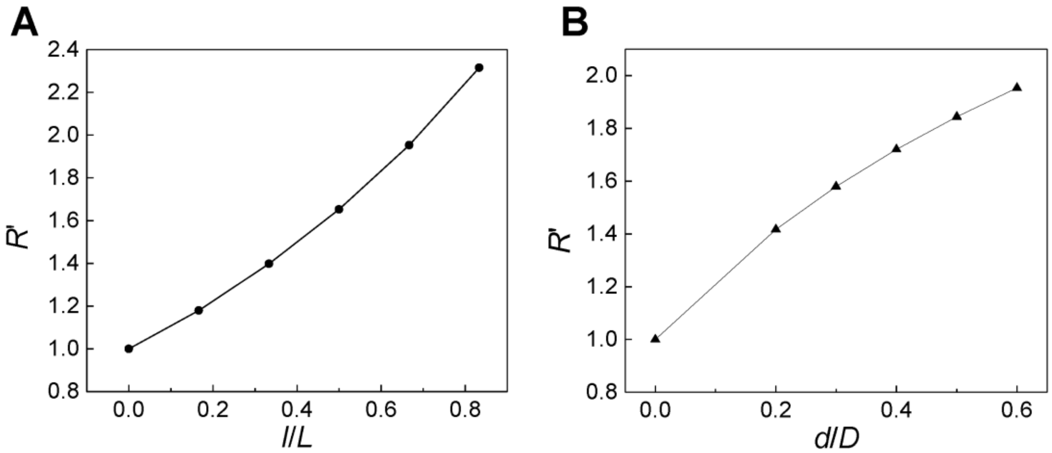

fig. S8. Effects of structural topography on the flow resistance parameter *R′*. (**A**) Variation of *R*' as a function of the length of cavity normalized by the total length of U-shaped island. (**B**) Variation of *R*' as a function of the width of cavity normalized by the width of U-shaped island.

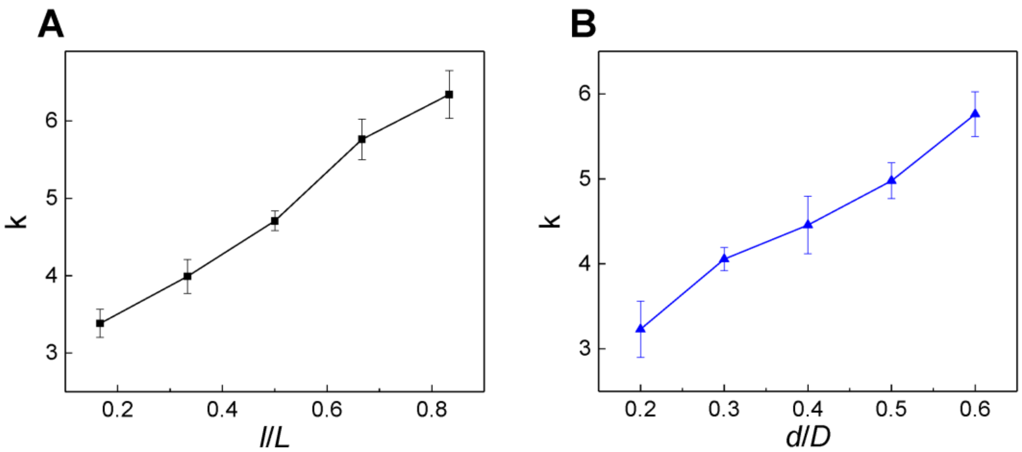

fig. S9. Effects of structural topography on the rectification coefficient *k*. (**A**) The variation of *k* on the liquid diodes as a function of the cavity length normalized by the total length of the U-shaped island. (**B**) The variation of *k* on the liquid diodes under a function of the cavity width normalized by the total width of the U-shaped island.

section S4. Characterization of macroscopic spreading dynamics on control surfaces

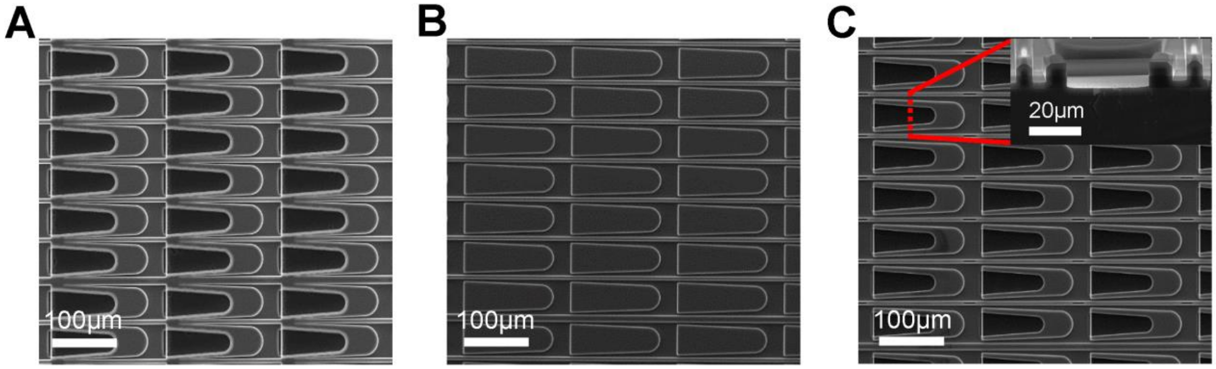

fig. S10. SEM characterization of control surfaces. (A) SEM image of control surface in which the side-channels are obstructed. (**B**) SEM image of control surface in which the cavities are obstructed. (**C**) SEM image of control surface with a straight sidewall in the inner side of the cavity. The inset image shows the magnified cross-sectional view of the cavity.

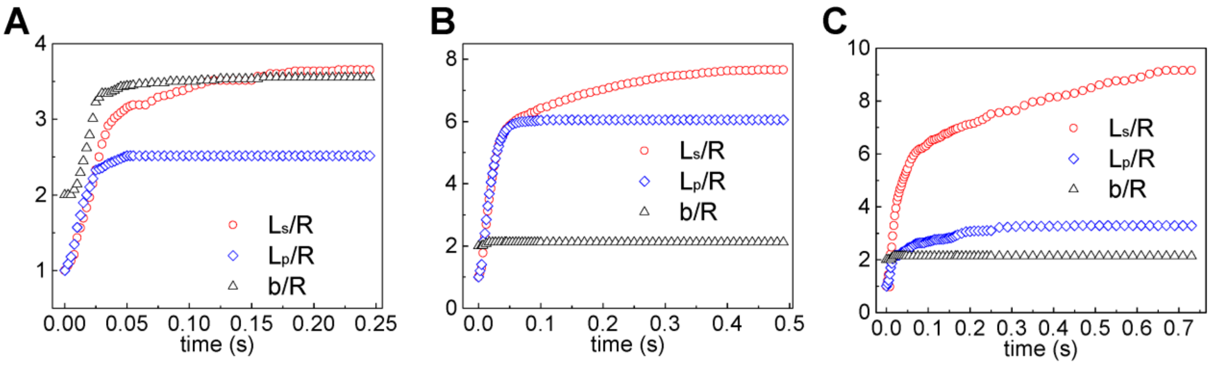

fig. S11. Spreading dynamics on the control surfaces. (**A**) Spreading dynamics on the control surface without the presence of side-channel. (**B**) Spreading dynamics on the control surface without the presence of cavity. (**C**) Spreading dynamics on the control surface without the presence of reentrant structure. Red circle and blue diamond symbols indicate the variation of the propagation distance of the contact line normalized by droplet radius R as a function of time in the spreading and the pinning directions, respectively. Black triangular symbols denote the variation of fluid width b normalized by droplet radius R.

section S5. Comparison of liquid self-transportation on various surfaces

To illustrate the advantages of our liquid diode, we benchmark the motion velocity and the range of transport with reported surfaces as shown in Fig. 4A and fig. S12. Surfaces with an imposed wetting gradient *(19, 25, 39-44)* are associated with a large spreading velocity (green area) and a short spreading distance owing to inherent conflicts in two parameters. On the contrary, asymmetrically structured surfaces *(7, 16, 20-22, 45, 46)* give rise to a long range of liquid self-transport and small velocity (blue area). It was recently reported that the peristome surface of pitcher plant (red triangular symbol) elegantly takes advantage of Taylor rise mechanism to attain fast liquid transport. Although this finding offers new insight for the design of directional surface, the structural and functional sophistication of this living organism requires stringent fabrication, and thus it is challenging to replicate and scale up to meet specific applications. Thus, we develop a new approach to create liquid diode (red circular symbol) for the continuous, long-range, and fast liquid self-propelling in the preferential direction.

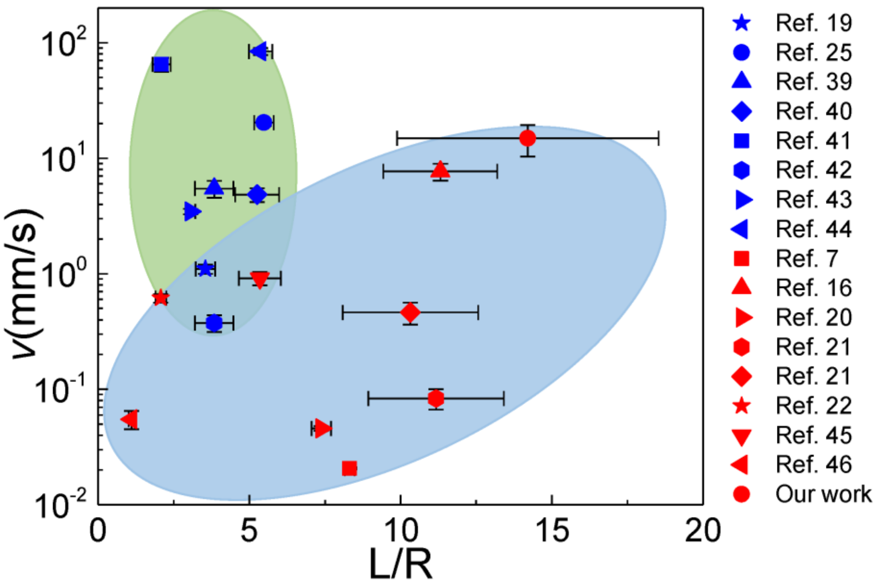

fig. S12. Comparison of transport performances among different surfaces. Green area and blue area indicate the surfaces with wettability gradients and asymmetric geometries, respectively. Especially, the red triangular symbol denotes the unidirectional liquid transport on the nature peristome of Pitcher plant. And red circular symbol represents the as-designed liquid diode.

section S6. Temperature gradient effect on the liquid diode

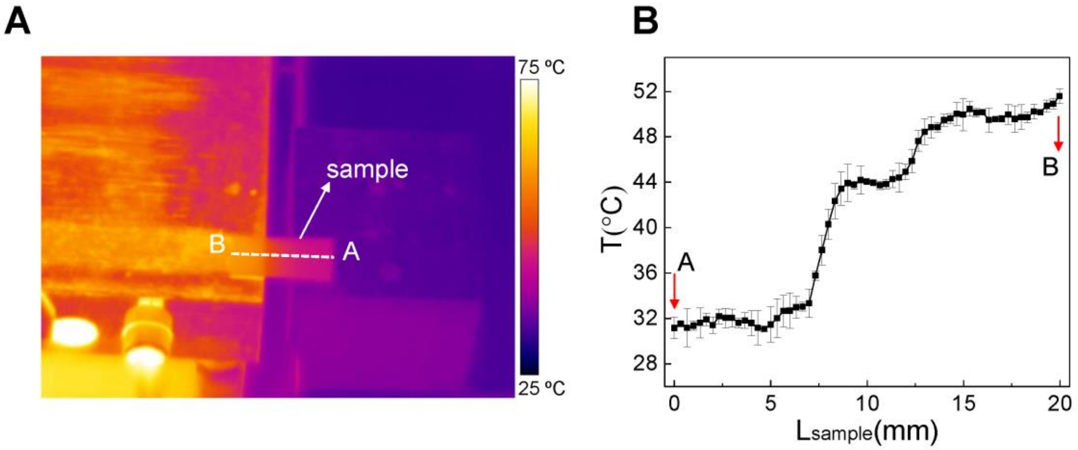

fig. S13. Effect of temperature gradient on the directional transport. (**A**) Infrared image showing the distribution of temperature on the liquid diode, which is imposed to an additional gradient temperature. (**B**) The variation of temperature on the liquid diode surface from A to B.

Legends for movies S1 to S6

movie S1. Unidirectional spreading of a single water droplet. When a deionized water droplet ~5 µl was deposited on the directional surface, it propagates towards one direction quickly, and gets stably pinned at the reverse direction. The width of the directional flow remained constant during spreading owing to the confinement effect of parallel fence structures.

movie S2. Microscopic wetting dynamics on the liquid diode. Approximately 1 µl water droplet was deposited on the liquid diode. Firstly, the liquid filament advances along the straight wall of fences and invades divergent side-channels under the capillary effect. Afterwards, the filament propagates around the inner sidewall of the cavity and gradually fills the cavity. Finally, the spreading liquid advances to the cavity in the downstream island, leading to the liquid propagation in a chain reaction manner.

movie S3. Corner flow in the divergent channel. As a water droplet containing 0.1µm fluorescent particles is placed in the mouth of a divergent channel, the liquid flows fast along all the available corners, and fills partially the entrance region of the channel. However, as flow continues along the corners, this accumulated liquid is depleted, indicating the role of the corner flow on the liquid transport.

movie S4. Hydraulic jump mechanism on the liquid diode. Approximately 1 µl water droplet was deposited on the liquid diode. As the semi-circular edge of the primary droplet is pinned, the liquid bulges up behind it thus resulting in a hydraulic jump forward like an avalanche.

movie S5. Directed water transportation on circular surface. The water was transported uni-directionally along the circular pathway to form a circular water rings. The band of water widened slightly owing the fabrication defect.

movie S6. Directed water transportation on spiral surface. The water was transported uni-directionally following a spiral pathway. The band of water widened slightly owing the fabrication defect.
